# Sex Differences in the Incidence of Sudden Cardiac Arrest/Death in Competitive Athletes: A Systematic Review and Meta-analysis

**DOI:** 10.1007/s40279-024-02163-5

**Published:** 2025-01-03

**Authors:** Lingxia Li, Solène Le Douairon Lahaye, Shuzhe Ding, Frédéric Schnell

**Affiliations:** 1 Sino-French Joint Research Center of Sport Science, College of Physical Education and Health, East China Normal University, Shanghai, China; 2 College of Physical Education and Health, East China Normal University, Shanghai, China; 3 Movement, Sport, and Health Science Laboratory (M2S Lab), University of Rennes 2, Rennes, France; 4 École Normale Supérieure de Rennes, Rennes, France; 5 Department of Sports Medicine, Pontchaillou Hospital, Rennes, France; 6 LTSI, INSERM, U1099, University of Rennes, Rennes, France; 7 CIC 1414, INSERM, University Hospital, University of Rennes, Rennes, France

## Abstract

**Background:**

Although many studies have demonstrated a lower incidence of sudden cardiac arrest or death (SCA/D) in female athletes than in male, there is limited understanding of the specific underlying causes.

**Objective:**

This systematic review aimed to assess the disparities in SCA/D incidence between male and female competitive athletes and explore the associated etiologies.

**Methods:**

A comprehensive search was conducted for retrospective and prospective studies examining SCA/D incidence in male and female athletes. Incidence and incidence rate ratios (IRRs) according to sex were evaluated.

**Results:**

Among the 16 studies analyzed, 1797 cases of SCA/D were observed; 1578 occurred in males (87.81%). Ages ranged from adolescent to adult. The incidence was 1.42/100,000 athlete-years (AY) in males (95% CI 0.97–2.09), and 0.32/100,000 AY in females (95% CI 0.17–0.59), resulting in an IRR of 5.55. When considering athletes aged ≤ 35 years, the incidence was 1.46/100,000 AY in males (95% CI 0.91–2.34) and 0.30/100,000 AY in females (95% CI 0.14–0.66), with an IRR of 5.47. The IRR was 5.13 (95% CI 3.94–6.67) for the most recent studies with athletes enrolled only after the year 2000, versus 6.02 (95% CI 4.59–7.90) for the remaining studies covering all observed years. Hypertrophic cardiomyopathy (HCM) was the predominant cause among males (45.12%), while congenital coronary anomalies were more prevalent in females (33.04%).

**Conclusion:**

The incidence of SCA/D in females was approximately 6 times lower than in males, with sex differences also in the leading causes of SCA/D. Understanding these discrepancies could lead to targeted strategies for the prevention of SCD in athletes.

**Registration number:**

(PROSPERO 2023 CRD42023432022)/05.07.2023.

**Supplementary Information:**

The online version contains supplementary material available at 10.1007/s40279-024-02163-5.

## Key Points

**Table:** 

The incidence of sudden cardiac arrest/death (SCA/D) in female competitive athletes was approximately 6 times lower than in male athletes.
There were sex differences in the most common causes of SCA/D. Hypertrophic cardiomyopathy was the most common cause in male athletes, while congenital coronary anomaly was most common in females.
Identifying the reasons for these differences may have implications for targeting the prevention of SCD in athletes.

## Introduction

Substantial evidence has shown that physical activity reaps healthy rewards [1]. Individuals engaging in regular physical activity have a lower risk of cardiovascular disease, cancer, and all-cause mortality [2]. However, acute and vigorous exercise may cause sudden cardiac death (SCD) in competitive athletes [3]. Relatively higher rates of SCD have been reported in collegiate athletes, Olympic athletes, triathletes, and cyclists [4–7]. Although the risk of SCD among athletes has decreased with the implementation of pre-participation screening programs, it remains relatively high in specific subgroups, notably in male athletes, Black athletes, and basketball players [8, 9]. Despite the methodological challenges, studies have consistently reported a lower risk of SCD in female athletes than in males [10]. However, little is known about the specific causes [11]. The recent *Lancet* Commission on SCD (2023) [12] highlighted this significantly higher risk of SCD in male athletes. Although the exact reasons remain unclear, the Commission proposed several possible factors, including the higher prevalence of coronary artery disease in young and middle-aged men compared with women, sex-based differences in nutrition and adherence to healthy lifestyles, and variations in triggers, autonomic modulators, and susceptibility to arrhythmias. They emphasized that while SCD in athletes is rare, it can broadly serve as a model for research on SCD more generally.

Existing knowledge on the incidence of SCD has been determined mainly based on studies conducted under specific circumstances using specific methodologies, often ignoring sex differences, or considering only one sex (predominantly males) [13–16]. Although Harmon et al. [17] summarized the incidence in both sexes via a state-of-the-art review, new studies were not included. Moreover, the estimated incidence, potential causes, and pathogenic mechanisms of SCD in athletes have yet to be effectively addressed and thoroughly and systematically evaluated.

Therefore, the present study aimed to perform a meta-analysis to evaluate the sex differences in the incidence of sudden cardiac arrest (SCA) and SCD in competitive athletes. Only studies with data on male and female athletes were included to avoid systematic bias in data aggregation.

## Methods

This systematic review and meta-analysis were conducted according to the Preferred Reporting Items for Systematic Reviews and Meta-Analysis (PRISMA) guidelines and recommendations [18]. They were registered in the International Prospective Register of Systematic Reviews (PROSPERO) (registration ID: CRD42023432022). Patient consent and ethics committee approval were not required.

### Search Strategy

Two investigators (LXL and FS) performed systematic database searches independently. PubMed, Embase, Scopus, SPORTDiscus, and Cochrane Library were searched. The heading terms ‘sudden cardiac death’ or (OR) ‘sudden cardiac arrest’ combined with (AND) ‘athletes’ were applied to identify the possible studies concerning SCA and/or SCD in athletes in published articles up to December 2023. Additionally, reference lists of eligible articles, relevant review articles, and meta-analysis reviews were manually searched to identify further relevant articles. Any disagreements were resolved by discussion with a third author (SLDL). The search strategies are available in the electronic supplementary material (ESM).

SCD was defined as a sudden unexpected death from a cardiac cause [19]. SCA was defined as an unexpected collapse due to a cardiac cause in which a cardiopulmonary resuscitation or an automated external defibrillator shock was performed in athletes who survived [19].

### Selection Criteria

The full text of identified articles was systematically assessed for eligibility after screening the title and abstract. The inclusion criteria for eligible studies in the meta-analysis were (1) written in English language, (2) published in peer-reviewed journals, (3) performed using prospective or retrospective cohort design, (4) observers included both sexes (a study was included when it reported events in both sexes but had 0 cases in one sex [usually in females]), (5) reported data including the incidence of SCA and/or SCD. Moreover, incidence rates that were not directly presented were generated based on existing data from the study; the rate was recalculated when the incidence was reported in a form other than per 100,000 athlete-years (AY); reported SCA/D that included causes unrelated to cardiac disease (such as trauma or heat stroke) were excluded, and the incidence was recalculated using the actual cases of SCA/D. The incidence rate per 100,000 AY was calculated as (number of events / total number of AY) × 100,000. When multiple reports were published on one dataset [4, 7, 8, 20–33], the study with a more extended observation period or more data was selected [4, 8, 30–33].

Studies were excluded when they (1) needed more sex-specific information, (2) had an absence of precise data to determine the incidence, and (3) had duplicated observational years of the populations. Supplementary materials were obtained when data were missing. These studies were discarded from subsequent analyses if the missing data were unavailable.

### Data Collection Process

Data collection was separately conducted by two authors (LXL and FS). The following information was extracted: (1) study characteristics (first author’s name, publication year, study country, study design, follow-up year, methods of case identification, and sample size of male and female participants); (2) participants’ information (onset age, type of events, and number of events in both sexes); and (3) clinical findings if available (e.g., autopsy reports).

### Quality Assessment of Eligible Studies

The Newcastle–Ottawa Quality Assessment scale [34] was used to assess the quality of each study included in the meta-analysis. The semi-quantitative principle of the star system was used in this scale. It consists of three elements with a total of eight items (the item “comparability of cohorts on the basis of design or analysis” was split into two sub-items, specifically “study controls for sex factor” and “study controls for other factor [e.g., type of sport and age]”). Each numbered item can be awarded a maximum of 1 star; thus, the total score was 9. Studies that scored 0–3, 4–6, and 7–9 were determined as low, medium, and high quality, respectively [35].

### Data Synthesis and Analysis

Incidence of SCA/D with corresponding 95% confidence intervals (95% CI) were calculated for both sexes. The incidence rate ratio (IRR) was used to compare the risk of SCA/D between the sexes. STATA (version 15.1; STATA Corp., USA) software was used for data analysis. A random-effects inverse-variance model was used for data pooling. Heterogeneity was assessed by I^2^ statistics. The magnitude of heterogeneity for I^2^ values < 30%, 30–70%, and > 70% represented low, medium, and high heterogeneity, respectively [14]. Subgroup analysis was performed with regards to age and study period. For age, we chose to use the cut-off value of 35 years of age, a widely accepted cut-off to differentiate young and veteran athletes. For the study period, we chose to differentiate the studies based on whether observation periods took place exclusively after the year 2000, in order to assess whether there has been any change, considering that women’s participation in competitive sports has grown significantly over the past decades.

## Results

### Study Selection

**Fig. 1:**
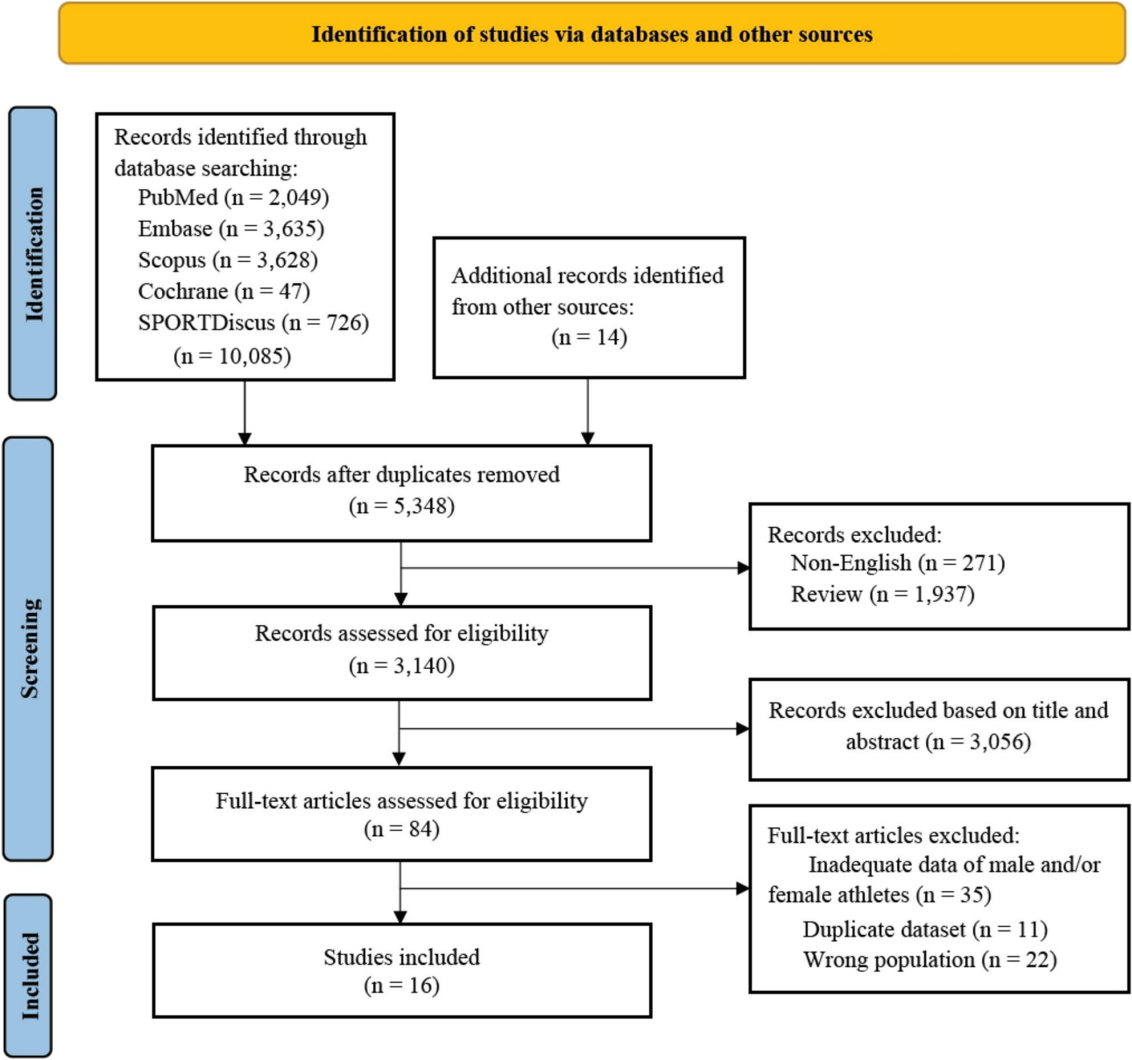
PRISMA flow diagram of the search process for studies examining the incidence of sudden cardiac arrest/death in male and female athletes

A total of 10,099 potential articles were identified from the combined systematic literature screen and other sources. After removing the duplicates, 5264 articles were excluded due to being non-English language studies (271), review articles (1937), and unrelated titles and abstracts (3056). Eighty-four studies were assessed for eligibility based on their full texts. Sixty-eight studies were excluded due to insufficient data of sex-specific information, duplicate datasets, and the wrong population. Sixteen studies were included in the meta-analysis (Fig. 1).

### Study Characteristics

Sixteen prospective or retrospective cohort studies were included, representing a combined sample of 268,729,566 participants per AY, including 154,928,991 AY males (57.65%) and 113,800,575 AY females (42.35%). Of the 1797 observed cases of SCA/D, 1578 occurred in male athletes (87.81%) and 219 in female athletes (12.19%). Ages ranged from adolescent to adult. The follow-up duration ranged from 2 years [36] to 32 years [4, 37]. The observational years ranged from 1979 [31] to 2022 [8]. Six studies assessed the events of both SCA and SCD [19, 32, 33, 38–40], while eight studies examined only SCD [8, 30, 31, 37, 41–44], and two studies examined only SCA [4, 36]. In four studies, SCA/D was found to occur only in male athletes, with 0 events in female athletes [30, 40, 43, 44].

Eleven studies covered various sports [8, 19, 30–32, 36–38, 41–43], while five studies focused on a single type of sport, including running (marathon and half-marathon) [33, 39, 40], triathlon [4], and soccer [44]. The countries studied were the United States (62.5%) [4, 8, 19, 30, 32, 33, 36, 37, 39, 42], Switzerland [41], Italy [31], France [40], Denmark [43], United Kingdom [44], and Japan [38]. Characteristics of all the 16 studies included are summarized in Table 1.

**Table 1:** Characteristics of the eligible studies

Study (year, country)	Years of follow-up (years)	Study design	Identification of the deaths	Sports category	Events	Occurrence (*n*)	Onset age (y)	Person-years of population	Incidence per 100,000 person-years (95% CI)
Petek et al. (2023, United States) [8]	20 years (2002–2022)	Retrospective and prospective	The NCAA resolution list, the Parent Heart Watch database or media report, NCAA insurance claims, and the National Center for Catastrophic Sports Injury Research database	Multiple	SCD	M = 119 F = 24	20	M = 5,158,412 F = 3,948,096	M: 2.31 (1.89–2.72) F: 0.61 (0.36–0.85)
Maron et al. (2016, United States) [37]	32 years (1980–2011)	Prospective	LexisNexis archival informational database, National Collegiate Athletic Association Memorial Resolutions List, news media, Internet search engine, reports from the US Consumer Product Safety Commission, records of the National Center for Catastrophic Sports Injury Research, and reports submitted to the Registry	Multiple	SCD	M = 747 F = 95	17.7 ± 4.8	M = 90,903,177 F = 74,802,240	M: 0.82 (0.76–0.88) F: 0.13 (0.10–0.15)
Kim et al. (2012, United States) [39]	10 years (2000–2010)	Prospective and retrospective	LexisNexis and Google search engine, local newspapers, official confirm, letter and mail to the next of kin, medical records, and questionnaires completed by the survivors and next of kin of nonsurvivors	Marathon and half-marathon	SCA/D	M = 51 F = 8	42 ± 13	M = 5,666,667 F = 5,189,333	M: 0.90 (0.65–1.15) F: 0.15 (0.05–0.26)
Corrado et al. (2003, Italy) [31]	21 years (1979–1999)	Prospective	Postmortem examination	Multiple	SCD	M = 47 F = 5	23.1 ± 7	M = 1,904,490 F = 464,100	M: 2.47 (1.76–3.17) F: 1.08 (0.13–2.02)
Harmon et al. (2016, United States) [19]	6 years (2007–2013)	Prospective	Parent Heart Watch records, systematic media search, and online queries	Multiple	SCA/D	M = 92 F = 12	14–18	M = 4,124,525 F = 2,850,115	M: 2.23 (1.77–2.69) F: 0.42 (0.18–0.66)
Harris et al. (2017, United States) [4]	32 years (1985–2016)	Retrospective and prospective	U.S. National registry of sudden death in athletes (which uses news media, Internet searches, LexisNexis archival database, and news clipping services) and USA Triathlon records	Triathlon	SCA	M = 9 F = 3	41.8 ± 8	M = 967,358 F = 742,199	M: 0.93 (0.32–1.54) F: 0.40 (0.00–0.86)
Holst et al. (2010, Denmark) [43]	7 years (2000–2006)	Retrospective	Death certificate, autopsy reports and selected hospital charts, and multiple registries	Multiple	SCD	M = 15 F = 0	15–35	M = 904,830 F = 334,663	M: 1.66 (0.82–2.50) F: 0.00 (0.00–0.00)
Gerardin et al. (2016, France) [40]	7 years (2006–2012)	Prospective	RACE Paris registry database records	Running	SCA/D	M = 12 F = 0	43 ± 10	M = 2,866,528 F = 716,632	M: 0.42 (0.18–0.66) F: 0.00 (0.00–0.00)
Maron et al. (2013, United States) [30]	26 years (1986–2011)	Prospective and retrospective	US National registry of sudden death in athletes	Multiple	SCD	M = 11 F = 0	12–18	M = 2,494,226 F = 1,945,935	M: 0.44 (0.18–0.70) F: 0.00 (0.00–0.00)
Malhotra et al. (2018, United Kingdom) [44]	21 years (1996–2016)	Retrospective	Registry from the English Football Association cardiac screening program (consists of a health questionnaire, physical examination, electrocardiography, and echocardiography)	Soccer	SCD	M = 8 F = 0	15–17	M = 112,433 F = 5918	M: 7.12 (2.18–12.05) F: 0.00 (0.00–0.00)
Peterson et al. (2021, United States) [32]	4 years (2014–2018)	Prospective	Registry from the National Center for Catastrophic Sports Injury Research in collaboration with national sports organizations	Multiple	SCA/D	M = 277 F = 54	11–29	M = 8,849,024 F = 6,568,862	M: 3.13 (2.76–3.50) F: 0.82 (0.60–1.04)
Roberts et al. (2013, United States) [33]	28 years (1982–2009)	Retrospective	Race records, personal communications, and newspaper accounts	Marathon	SCA/D	M = 13 F = 1	28–60	M = 379,863 F = 168,227	M: 3.42 (1.56–5.28) F: 0.59 (0.00–1.76)
Suzuki-Yamanaka et al. (2022, Japan) [38]	10 years (2009–2018)	Retrospective	Insurance claim data of fatalities and severe injuries from JSC Injury and Accident Mutual Aid Benefit System for disability or death compensations	Multiple	SCA/D	M = 51 F = 4	15–18	M = 9,607,259 F = 4,419,729	M: 0.53 (0.39–0.68) F: 0.09 (0.00–0.18)
Toresdahl et al. (2014, United States) [36]	2 years (2009–2011)	Prospective	National Registry for AED use in sports, and email and phone interview	Multiple	SCA	M = 16 F = 2	13–19	M = 924,538 F = 652,828	M: 1.73 (0.88–2.58) F: 0.31 (0.00–0.73)
Van Camp et al. (1995, United States) [42]	11 years (1983–1993)	Retrospective	Registry from the National Center for Catastrophic Sports Injury Research, and national newspaper clipping service	Multiple	SCD	M = 92 F = 8	13–24	M = 19,550,662 F = 10,492,917	M: 0.47 (0.37–0.57) F: 0.08 (0.02–0.13)
Asatryan et al. (2017, Switzerland) [41]	12 years (1999–2010)	Retrospective	Forensic autopsy reports	Multiple	SCD	M = 18 F = 3	10–39	M = 514,999 F = 498,778	M: 3.50 (1.88–5.11) F: 0.60 (0.00–1.28)

*AED* automated external defibrillators, *F* female, *M* male, *NCAA* National Collegiate Athletic Association, *SCA* sudden cardiac arrest with survival, *SCD* sudden cardiac death

### Quality Assessment of the Eligible Studies

All the eligible papers obtained at least seven stars. Six studies scored 7 (37.50%), seven studies scored 8 (43.75%), and three scored 9 (18.75%). All these papers were considered to have high methodological quality (Table 2).

**Table 2:** Results of quality assessment using the Newcastle–Ottawa Scale for observational cohort studies

Study (year, country)	Selection				Comparability		Outcome			Scores
	Representative of the exposed cohort	Selection of the non-exposed cohort	Ascertainment of exposure	Demonstration that outcome of interest was not present at start of study	Comparability of cohorts on the basis of the design or analysis		Assessment of outcome	Follow-up long enough for outcome to occur	Adequate follow up of cohorts	
					Study controls for sex factor	Study controls for other factor				
Petek et al. (2023, United States) [8]	☆	☆	☆	☆	☆	☆	☆	☆	☆	9
Maron et al. (2016, United States) [37]	☆	☆	☆	☆	☆	☆	☆	☆	☆	9
Kim et al. (2012, United States) [39]	☆	☆	☆	☆	☆	☆	☆	☆	☆	9
Asatryan et al. (2017, Switzerland) [41]	☆	☆	☆		☆	☆	☆	☆	☆	8
Corrado et al. (2003, Italy) [31]	☆	☆	☆	☆	☆		☆	☆	☆	8
Harmon et al. (2016, United States) [19]	☆	☆	☆	☆	☆		☆	☆	☆	8
Harris et al. (2017, United States) [4]	☆	☆	☆		☆	☆	☆	☆	☆	8
Holst et al. (2010, Denmark) [43]	☆	☆	☆		☆	☆	☆	☆	☆	8
Gerardin et al. (2016, France) [40]	☆	☆	☆		☆	☆	☆	☆	☆	8
Maron et al. (2013, United States) [30]	☆	☆	☆		☆	☆	☆	☆	☆	8
Malhotra et al. (2018, United Kingdom) [44]	☆	☆	☆		☆		☆	☆	☆	7
Peterson et al. (2021, United States) [32]	☆	☆	☆	☆	☆		☆		☆	7
Roberts et al. (2013, United States) [33]	☆	☆	☆		☆		☆	☆	☆	7
Suzuki-Yamanaka et al. (2022, Japan) [38]	☆	☆	☆		☆		☆	☆	☆	7
Toresdahl et al. (2014, United States) [36]	☆	☆	☆	☆	☆		☆		☆	7
Van Camp et al. (1995, United States) [42]	☆	☆	☆		☆		☆	☆	☆	7

Each star corresponds to one score. A follow-up period of ≥ 5 years was deemed sufficient for outcomes to manifest and receive one score. Studies examining additional factors influencing sex differences, such as sports, ethics, etiology, etc., with fewer than two were deemed insufficient for addressing these factors and were not scored

### Synthesis of Results

**Fig. 2:**
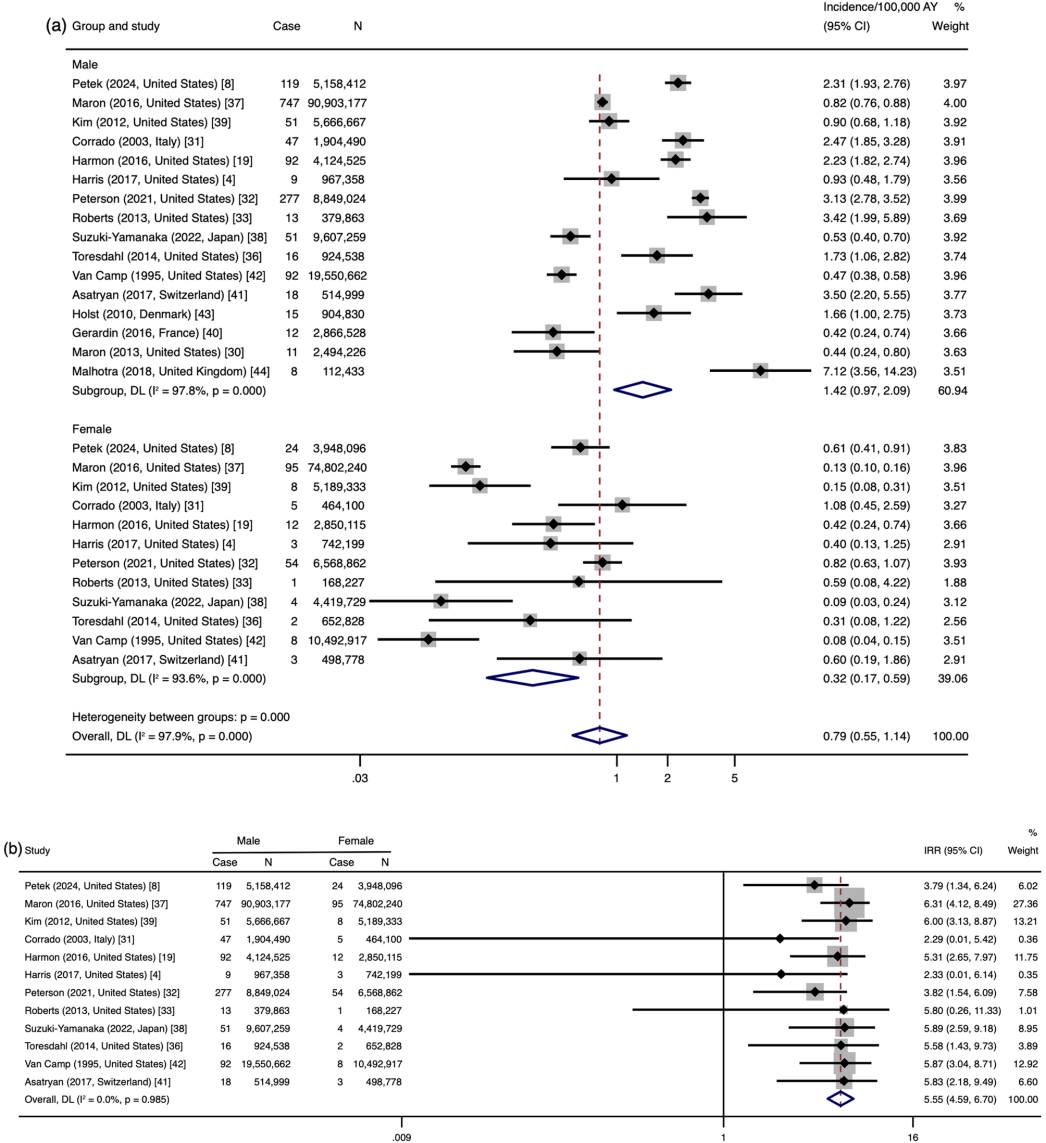
Incidence (**a**) and incidence rate ratio (IRR) (**b**) of sudden cardiac arrest/death in male and female athletes (*n* = 16 studies) *AY* athlete-years, *Case* the number of sudden cardiac arrests/deaths, *DL* data analyzed by the random-effects of DerSimonian and Laird method, *IRR* incidence rate ratio, *N* the athlete-years of participants

The initial random effects pooled analysis of the 16 studies found that the incidence of SCA/D in male athletes varied from 0.42 to 7.12 per 100,000 AY, while the incidence varied from 0.00 to 1.08 per 100,000 AY in female athletes. The estimated incidence was 1.42/100,000 AY in male athletes (95% CI 0.97–2.09) and 0.32/100,000 AY in female athletes (95% CI 0.17–0.59). There was high heterogeneity among the studies (male: *I*^2^ = 97.8%; female: *I*^2^ = 93.6%) (Fig. 2a). The IRR between the sexes was 5.55 (95% CI 4.59–6.70) and the heterogeneity was low (*I*^2^ = 0.0%) (Fig. 2b).

### Subgroup Analysis

#### Synthesis of Studies with Subjects ≤ 35 Years of Age

**Fig. 3:**
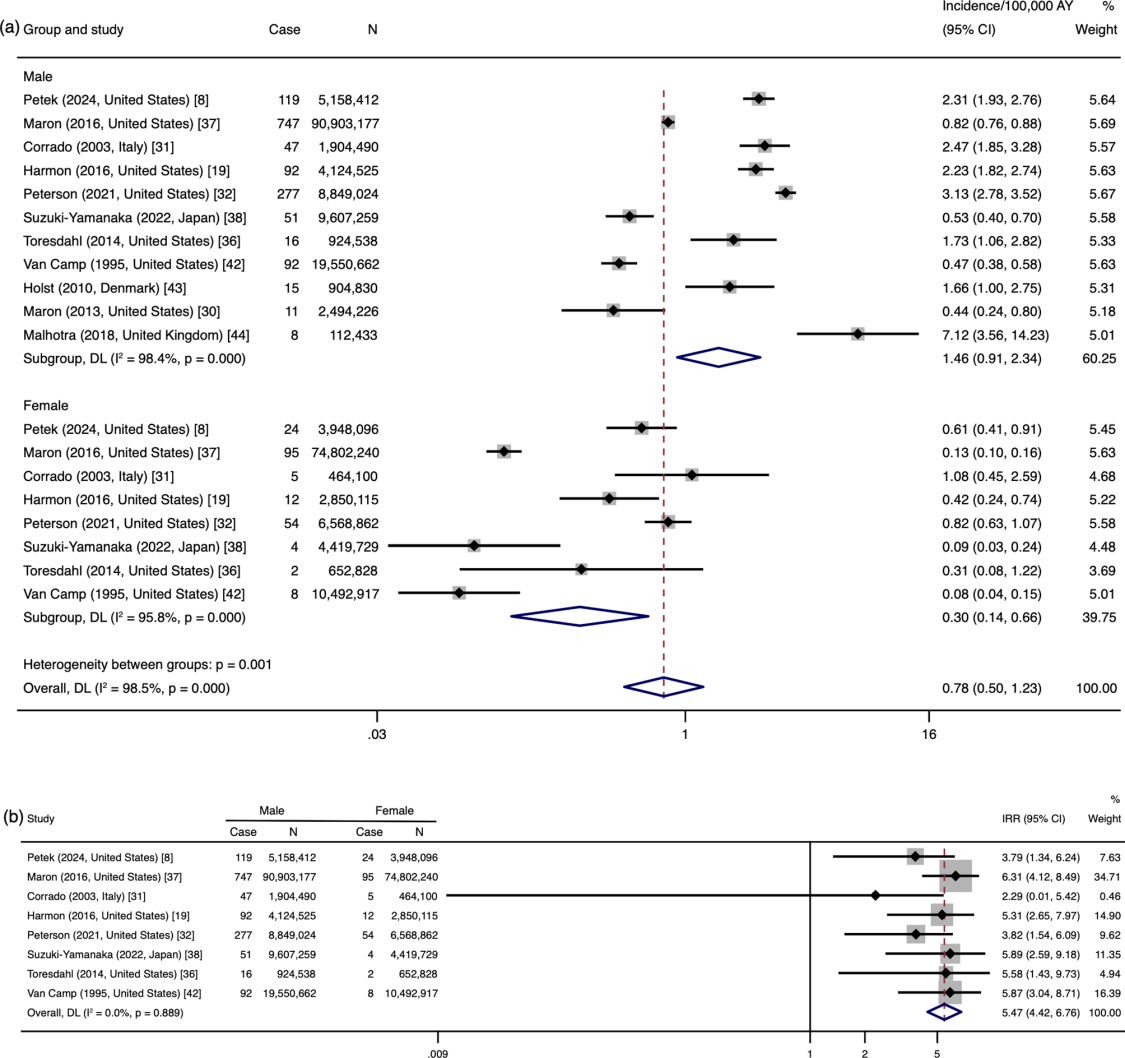
Incidence (**a**) and incidence rate ratio (IRR) (**b**) of sudden cardiac arrest/death in male and female athletes aged ≤ 35 years (*n* = 11 studies). *AY* athlete-years, *Case* the number of sudden cardiac arrests/deaths, *DL* data analyzed by the random-effects of DerSimonian and Laird method, *IRR* incidence rate ratio, *N* the athlete-years of participants

Subgroup data synthesis of the 11 studies [8, 19, 30–32, 36–38, 42–44] in which the age of the subjects was ≤ 35 years showed that the estimated incidence in male athletes was 1.46/100,000 AY (95% CI 0.91–2.34) and it was 0.30/100,000 AY (95% CI 0.14–0.66) in female athletes. There was high heterogeneity among the studies (male: *I*^2^ = 98.4%; female: *I*^2^ = 95.8%) (Fig. 3a). The IRR between the sexes in athletes aged ≤ 35 years was 5.47 (95% CI 4.42–6.76), and the heterogeneity was low (*I*^2^ = 0.0%) (Fig. 3b).

#### Synthesis of Studies with Observation Periods Occurring After the Year 2000

**Fig. 4:**
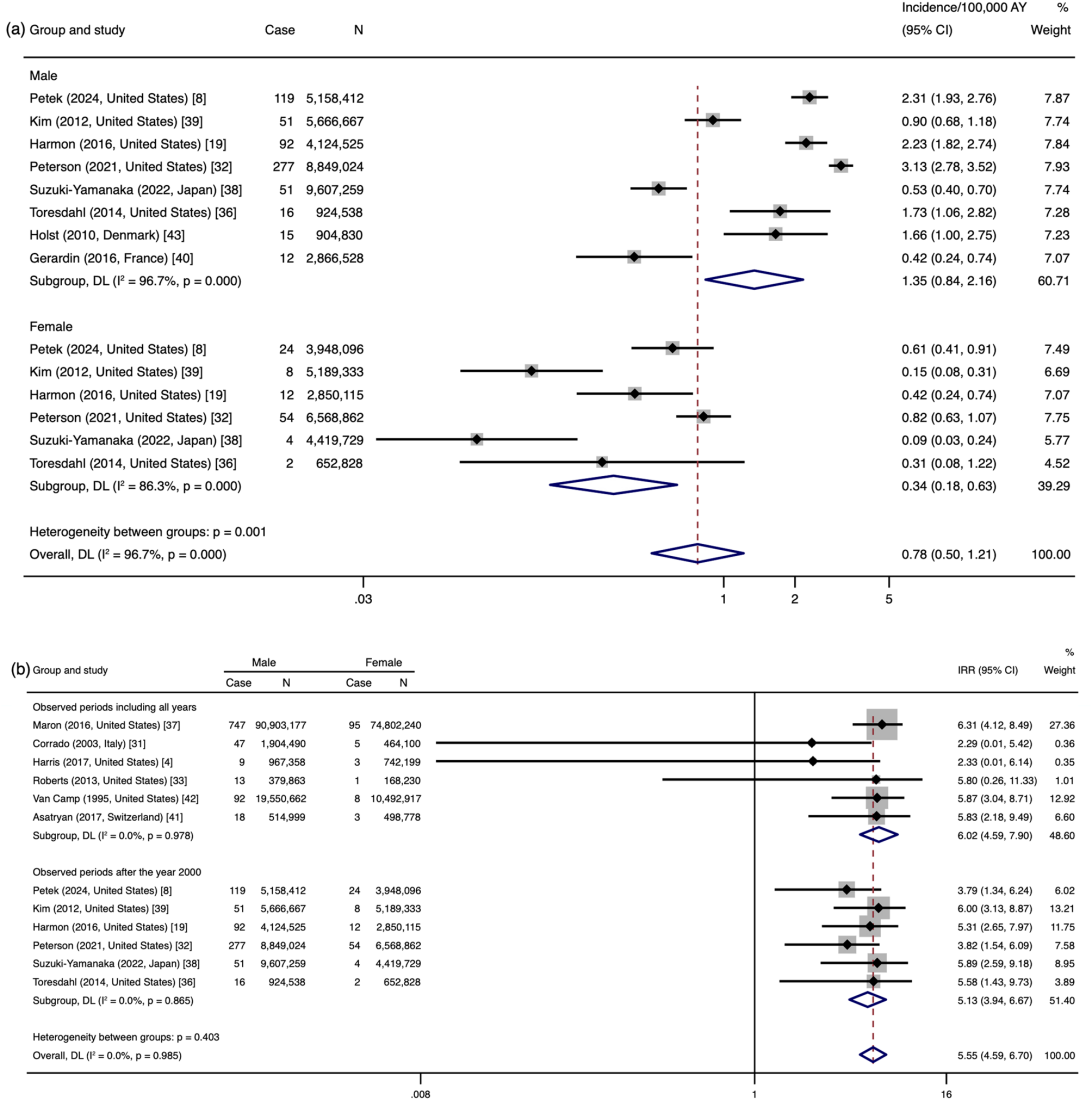
Incidence of sudden cardiac arrest/death in male and female athletes observed after the year 2000 (*n* = 8 studies) (**a**), and incidence rate ratio (IRR) of sudden cardiac arrest/death in male and female athletes observed after the year 2000 or observed in all years before and after the year 2000 (**b**). *AY* athlete-years, *Case* the number of sudden cardiac arrests/deaths, *DL* data analyzed by the random-effects of DerSimonian and Laird method, *IRR* incidence rate ratio, *N* the athlete-years of participants

Subgroup data analysis of the eight studies [8, 19, 32, 36, 38–40, 43] in which the observed periods were after the year 2000 revealed that the incidence was 1.35/100,000 in males (95% CI 0.84–2.16) and 0.34/100,000 in females (95% CI 0.18–0.63). There was high heterogeneity among the studies (males: *I*^2^ = 96.7%; females: *I*^2^ = 86.3%) (Fig. 4a). The IRR in studies with observed periods after the year 2000 was 5.13 (95% CI 3.94–6.67), while the IRR in the remaining studies was 6.02 (95% CI 4.59–7.90); the heterogeneity was low (*I*^2^ = 0.0%) (Fig. 4b).

### Etiologies of the SCA/D

**Fig. 5:**
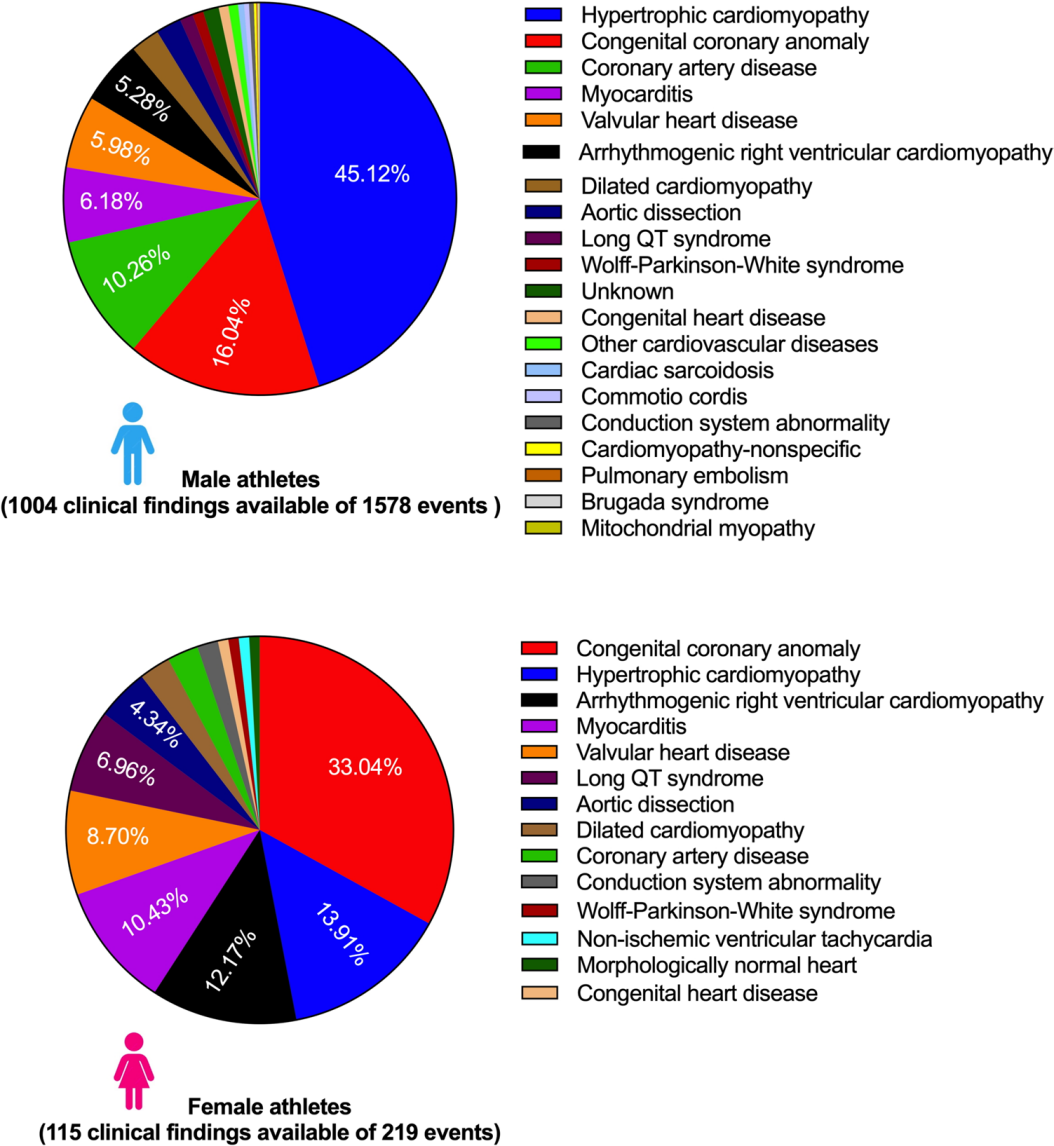
Clinical findings of the cause of sudden cardiac arrest/death in male and female athletes

From the 16 studies included, the etiologies of the SCA/D were reported in 1119 cases; they were reported in 63.62% of male athletes (1004/1578) and 52.51% (115/219) of female athletes. The most common cause of SCA/D in male athletes was hypertrophic cardiomyopathy (HCM) (453/1004 [45.12%]), followed by congenital coronary anomaly (161/1004 [16.04%]), and coronary artery disease (CAD) (103/1004 [10.26%]). The most common cause of SCA/D in females was congenital coronary anomaly (38/115 [33.04%]), followed by HCM (16/115 [13.91%]) and arrhythmogenic right ventricular cardiomyopathy (ARVC) (14/115 [12.17%]) (Fig. 5).

## Discussion

This meta-analysis assessed the sex differences in the incidence of SCA/D in competitive athletes. Previous studies have explored the incidence of SCA or SCD in athletes under specific circumstances using specific methodologies, but they often ignored the sex differences. Of the 16 studies included in the present meta-analysis, we found that (1) the incidence in female athletes was 5.55 times lower than in males; (2) considering the athletes ≤ 35 years of age, the incidence in females was 5.47 times lower than in males; (3) the IRR in studies of observed periods after the year 2000 was 5.13 (95% CI 3.94–6.67); and (4) the causes of SCA/D were different as regards sex: in male athletes the most common cause was HCM, while in females it was congenital coronary anomaly.

### Sex Differences in Incidence of SCA/D

Women represent an increasing proportion of sports participants. However, there is still a paucity of original data on the incidence of SCA/D in female athletes [45]. Consistent with previous studies [10], the present study found that female athletes had an approximately 6 times lower estimated incidence of SCA/D than males. Considering the athletes ≤ 35 years of age, the estimated incidence was 5.47 times lower in females than in males. Studies of young Swedish orienteers between 1979 and 1992 found that of 16 cases of unexpected SCD, 15 were male athletes, and only one was a female athlete [46]; a large regional registry in the UK found that of the 748 cases of SCD in people who exercise regularly, only 13% were women, demonstrating a lower incidence of SCD in female athletes [11]. The average annual incidence from European registries of sports-related SCA in females was 0.19/100,000, more than ten times lower than in male athletes, despite similar subject characteristics [45]. A prospective study conducted in France between 2005 and 2010 found that the estimated incidence rate in female athletes was only 0.51/100,000 AY (95% CI 0.34–0.68), compared with 10.1/100,000 AY (95% CI 9.3–10.8) in male athletes [47]. One explanation frequently provided is the lower prevalence of females in the athlete population. However, previous studies have provided evidence that the lower incidence of SCDs in women cannot be attributed simply to lower participation rates [48]. Indeed, in the prospective Race Paris registry of cardiac events in long-distance running, collecting data on 1,000,000 participants over 10 years, only one life-threatening cardiac event occurred in a female athlete, though females accounted for 22% of all runners [49].

In the subgroup analysis on the studies covering only the periods after the year 2000, the IRR was 5.13 (95% CI 3.94–6.67), while the IRR in the remaining studies was 6.02 (95% CI 4.59–7.90). A previous publication reported that the incidence of SCD increased at a rate of 6% per year from 1994 to 2006 compared with 1980 to 1993, and that the proportion of all deaths in females has increased over time [3]. Another study showed that the incidence of sports-related SCA remained relatively stable over time, with a decrease of SCD only due to a major improvement in on-field resuscitation (basic life support and public automated external defibrillator use) [50]. However, in this meta-analysis, there were several challenges in comparing incidence rates across trials since the estimates varied depending on the age of the athletes, the source of the sampling population, the sports activity, and the definition of sudden death [51].

### Sex Differences in Etiologies of SCA/D

In male athletes, the most common cause of SCA/D was HCM (45.12%), followed by congenital coronary anomaly (16.04%), CAD (10.26%), myocarditis (6.2%), valvular heart disease (5.98%), and ARVC (5.28%). This is consistent with the literature in a population of young athletes [52, 53]. In female athletes, CAD was considerably less common and the other etiologies were encountered in a different order of frequency. Congenital coronary anomaly was the most frequent (33.04%), followed by HCM (13.91%), ARVC (12.17%), myocarditis (10.43%), and valvular heart disease (8.7%). The difference in the prevalence of CAD is the same as in the general population of non-athletes. Women generally develop CAD at an older age, which is linked to the hormonal protection provided by estrogen in pre-menopausal women [54]. It was also demonstrated that CAD mortality was up to five times higher in middle-aged men than women [55]. Regarding the other etiologies encountered, there is no difference in the prevalence of HCM as regard to sex; nevertheless, male sex is a risk factor for SCA/D in HCM [56]. The explanation is still unclear; some authors have suggested that the difference may be partly explained by the greater intensity of training and participation in competition in male subjects; however, this explanation is not convincing in our population of athletes [57]. Females undergo significant cardiac remodeling compared with sedentary controls but rarely reach absolute left ventricular dimensions or wall thickness that overlap with dilated cardiomyopathy or HCM [58]. Whether this might have any influence on the risk of SCD is still unclear. Therefore, identifying the protective mechanism of the cardiovascular system in female athletes may help in finding a way to mitigate the risk of SCD in athletes.

### Limitations

The narrow inclusion criteria (each study included data relevant to both sexes) reduced the risk of bias. This might explain why despite the high heterogeneity in the incidence of SCA/D among male and female athletes, the heterogeneity in the IRR between sexes was low, which reinforces the reliability of our study’s conclusions. However, this was a limitation at the same time. Several studies of SCA/D in athletes had large samples and data but were excluded due to a lack of information on sex-specific information [53]. This reduced the amount of data included in the meta-analysis and weakened the evidence base. In addition, data synthesis revealed high heterogeneity within the group, suggesting some moderators influenced sex-specific variability. However, due to the limited number of studies included in this meta-analysis, only sub-group analyses of athletes aged ≤ 35 years and the observational periods after the year 2000 were conducted, and it was not possible to examine factors such as ethnicity, type of sports, and sports levels in sex differences.

Another limitation of our study relates to the narrow inclusion criteria, as we chose to focus on the occurrence of SCA/D in competitive athletes, excluding recreational sports. We recognize that numerous studies have shown SCA/D to be more frequent among recreational sports participants compared with competitive athletes [59, 60]. However, the sex discrepancies in SCA/D occurrence appear even more pronounced in recreational sports. A previous study reported that the incidence in women was up to 30 times lower in the middle-aged group [48].

Finally, a limitation of the study is that all included research was conducted in Western countries, with no data available from low- and middle-income countries. This is a significant concern, as noted by the Lancet SCD Commission [12], because it limits our understanding of SCD and the potential for effective prevention in these regions.

### Perspectives

Since, in most patients, SCA occurs without warning symptoms [61], cardiovascular pre-participation screening attempts to identify pre-existing cardiac abnormalities, ensuring optimal management and thereby reducing the potential for adverse events and sports-related fatalities [62]. The incidence of SCD in young competitive athletes has substantially decreased in the Veneto region of Italy since the implementation of systematic screening [9]. However, the low incidence in female athletes challenges the cost effectiveness of screening in female athletes [63]. A better comprehension of the sex differences in the rate of SCA/D might help us to perform a better pre-participation screening strategy in both males and females [10].

## Conclusion

The meta-analysis highlights significant sex differences in the incidence of SCA/D among competitive athletes, with female athletes showing an approximately six times lower incidence compared with males, with low heterogeneity observed. Understanding the underlying reasons for these differences could be crucial for improving targeted SCD prevention strategies.

## Supplementary Information

Below is the link to the electronic supplementary material.

Supplementary file1 (PDF 135 KB)

## Declarations

### Data Availability

Data used in the present study such as data extraction templates, forms and analysis will be made available upon reasonable request to the corresponding author.
