# Supplementary material for: Sex Differences in the Incidence of Sudden Cardiac Arrest/Death in Competitive Athletes: A Systematic Review and Meta-analysis
Source: Sports Med. 2025 Jan 3;55(3):697–712. doi: 10.1007/s40279-024-02163-5 (PMC11985649; doi:10.1007/s40279-024-02163-5)
Supplement: Supplementary file 1 — Supplementary file1 (PDF 135 KB) [file 40279_2024_2163_MOESM1_ESM.pdf]

Sex differences in the incidence of sudden cardiac arrest/death in competitive athletes: A systematic review and meta-analysis

Short title: Sex differences in the incidence of SCA/D in competitive athletes

Sports Medicine

Lingxia Li [1,2,3], Solène Le Douairon Lahaye [3,4], Shuzhe Ding [2], Frédéric Schnell [5,6,7*]

[1] Sino-French Joint Research Center of Sport Science, College of Physical Education and Health, East China Normal University, Shanghai, China

[2] College of Physical Education and Health, East China Normal University, Shanghai, China

[3] Movement, Sport, and Health Science Laboratory (M2S Lab), University of Rennes 2, Rennes, France

[4] École Normale Supérieure de Rennes, Rennes, France

[5] Department of Sports Medicine, Pontchaillou Hospital, Rennes, France

[6] LTSI, INSERM, U1099, University of Rennes, Rennes, France

[7] CIC 1414, INSERM, University Hospital, University of Rennes, Rennes, France

*Corresponding author:

Prof. Frédéric Schnell

frederic.schnell@chu-rennes.fr

Search strategies

- ✧ Keywords: athletes; sudden cardiac arrest/death
- ✧ Searching date: 2022.12.28-2023.06.15

PubMed   Results: 2049

#1 "Athletes"[MeSH Terms] OR "Athletes"[Title/Abstract]

#2 "death, sudden, cardiac"[MeSH Terms] OR "sudden cardiac death" [Title/Abstract] OR "sudden death"[Title/Abstract] OR "cardiac death"[Title/Abstract] OR "sudden cardiac arrest"[Title/Abstract] OR "cardiac arrest"[Title/Abstract]

#3 #1 AND #2

Embase   Results: 3635

#1 athletes'/exp OR athletes

#2 'sudden cardiac death'/exp OR 'sudden cardiac death' OR (sudden AND ('cardiac'/exp OR cardiac) AND ('death'/exp OR death)) OR 'cardiac death':ab,ti OR 'sudden death':ab,ti OR 'sudden cardiac arrest':ab,ti OR 'cardiac arrest':ab,ti

#3 #1 AND #2

Scopus   Results: 3628

#1 TITLE-ABS-KEY (athletes)

#2 (TITLE-ABS-KEY (sudden AND cardiac AND death) OR TITLE-ABS-KEY (sudden AND death) OR TITLE-ABS-KEY (cardiac AND death) OR TITLE-ABS-KEY (sudden AND cardiac AND arrest) OR TITLE-ABS-KEY (cardiac AND arrest))

#3 #1 AND #2

SPORT Discus   Results: 726

#1 TI athletes OR AB athletes

#2 TI sudden cardiac death OR AB sudden cardiac death OR TI sudden death OR AB sudden death OR TI cardiac death OR AB cardiac death OR TI sudden cardiac arrest OR AB sudden cardiac arrest OR TI cardiac arrest OR AB cardiac arrest

#3 #1 AND #2

Cochrane   Results: 47

#1 MeSH descriptor: [Athletes] explode all trees

#2 (athletes):ti,ab,kw

#3 #1 OR #2

#4 MeSH descriptor: [Death, Sudden, Cardiac] explode all trees

#5 (sudden cardiac death):ti,ab,kw OR (cardiac death):ti,ab,kw OR (sudden death):ti,ab,kw OR (sudden cardiac arrest):ti,ab,kw OR (cardiac arrest):ti,ab,kw

#6 #4 OR #5

#7 #3 AND #6
